# Composition formulas of solid-solution alloys derived from chemical-short-range orders

**DOI:** 10.1038/s41598-022-06893-2

**Published:** 2022-02-24

**Authors:** Zhuang Li, Dandan Dong, Lei Zhang, Shuang Zhang, Qing Wang, Chuang Dong

**Affiliations:** 1 Key Laboratory for Materials Modification by Laser, Ion and Electron Beam (Dalian University of Technology), Ministry of Education, Dalian, 116024 China; 2 College of Physical Science and Technology, Dalian University, Dalian, 116622 China; 3 Science and Technology on Surface Physics and Chemistry Laboratory, Mianyang, 621907 China; 4 School of Materials Science and Engineering, Dalian Jiaotong University, Dalian, 116028 China

**Keywords:** Condensed-matter physics, Materials science, Metals and alloys

## Abstract

Solid solutions are the basis for most industrial alloys. However, the relationships between their characteristic short-range orders and chemical compositions have not been established. The present work combines Cowley parameter *α* with our cluster-plus-glue-atom model to accurately derive the chemical units of binary solid-solution alloys of face-centered cubic type. The chemical unit carries information on atomic structure and chemical composition, which explains prevailing industrial alloys. For example, chemical units in Cu_68.9_Zn_31.1_ alloy with *α*_1_ = − 0.137 are formulated as [Zn-Cu_10_Zn_2_]Zn_2_Cu_2_ and [Zn-Cu_10_Zn_2_]Zn_3_Cu_1_, with 64.0–70.0 wt% Cu corresponding to the most widely used cartridge brass C26000 (68.5–71.5 Cu). This work answers the long-standing question on the composition origin of solid-solution-based industrial alloys, by tracing to the molecule-like chemical units implied in chemical short-range ordering in solid solutions.

## Introduction

In one of the early review on solid solutions in 1925, Bruni^1^ raised a preliminary question: does the chemical molecule continue to exist in the crystalline state? This question looks quite naive at present but must be answered in his time as most of the metals are based on solid solutions and they all have specific chemical compositions, just like any molecular substance whose chemistry is contained in the molecular structure. The first results of X-ray analyses by Bragg^2^ answered this question in the negative, by affirming that within the crystal edifice only atoms exist and the molecule vanishes into the lattice. However, the structural origin of chemical compositions of industrial alloys remains open. The key to understanding the composition mystery must lie in the structure of solid solutions, which has been a hot topic in the early twentieth century. Bragg and Williams were among the first to propose a statistical model that considers the order and disorder in solid solutions as a co-operative long-range phenomenon^3^. This model was then extended to a more elaborated theory by Bethe^4^, assuming the short-range interaction in nearest neighborhood. The long- and short-range orders are well unified in Cowley’s^5^ short-range order parameters *α*_i_, expressing the interaction of a given atom A with the atoms of the *i*th shell of atoms surrounding it:

1 $$\alpha_{{\text{i}}} = 1 - \frac{{n_{i} }}{{m_{B} c_{i} }}$$

where *n*_*i*_ is the number of B atoms among the *c*_*i*_ atoms of the *i*th shell, and *m*_B_ is the mole fraction of B atoms in A–B binary alloy. Equations for the long-range order parameter of Bragg and Williams are obtained by considering the limiting case of *i* very large. Since then it is well recognized that short-range ordering is the major structural feature of solid solutions.

In an effort to explore the composition origin implied in such ordered and disordered local structures, our team has been engaged in developing a so-called cluster-plus-glue-atom model^6–8^ which simplifies any short-range order into a local unit covering a nearest-neighbor cluster plus a few next-neighbor glue atoms, expressed in cluster formula form as [cluster](glue atoms). This structural unit, showing charge neutrality and mean density following Friedel oscillation^9^, resembles in many ways chemical molecules and henceforth is termed ‘chemical unit’^7^. The only difference from common concept of molecule lies in the way the chemical units are separated: instead of relatively weak inter-molecular forces between molecules, here the chemical units are linked by chemical bonding. We have shown by analyzing many industrial alloys that popular alloys are all based on simple cluster-plus-glue-atom formulas, such as [Zn-Cu_12_]Zn_4_ for Cu-30Zn, [Ni-Fe_12_]Cr_2_(Ni,Nb,Ti) for marageing stainless steel Custom465, etc.^7^.

However, despite of the proved capacity of the cluster-plus-glue-atom model in interpreting composition origins of alloys, there is an obvious gap between the idealized formulas (e.g., the nearest neighbors are always fully occupied by solvent atoms such as [Zn-Cu_12_]Zn_4_) and the real chemical short-range ordering (the nearest neighbors are always mixed-occupied) that can be measured, for example using parameter *α*_i_. The *α*_i_ parameter describes the statistical deviation from the average alloy composition in each redial shell. The composition deviation appears most prominently in the first and second nearest neighbors, which agrees perfectly with the picture of the cluster-plus-glue-atom model that covers also the same radial range. The present work is our first attempt to fill in the gap, by showing how to relate the measurable parameters *α*_i_, within the framework of the cluster-plus-glue-atom model, to the construction of composition formulas of typical binary solid solution alloys with face-centered cubic (FCC) structure.

## Theoretical methods

**Figure 1:**
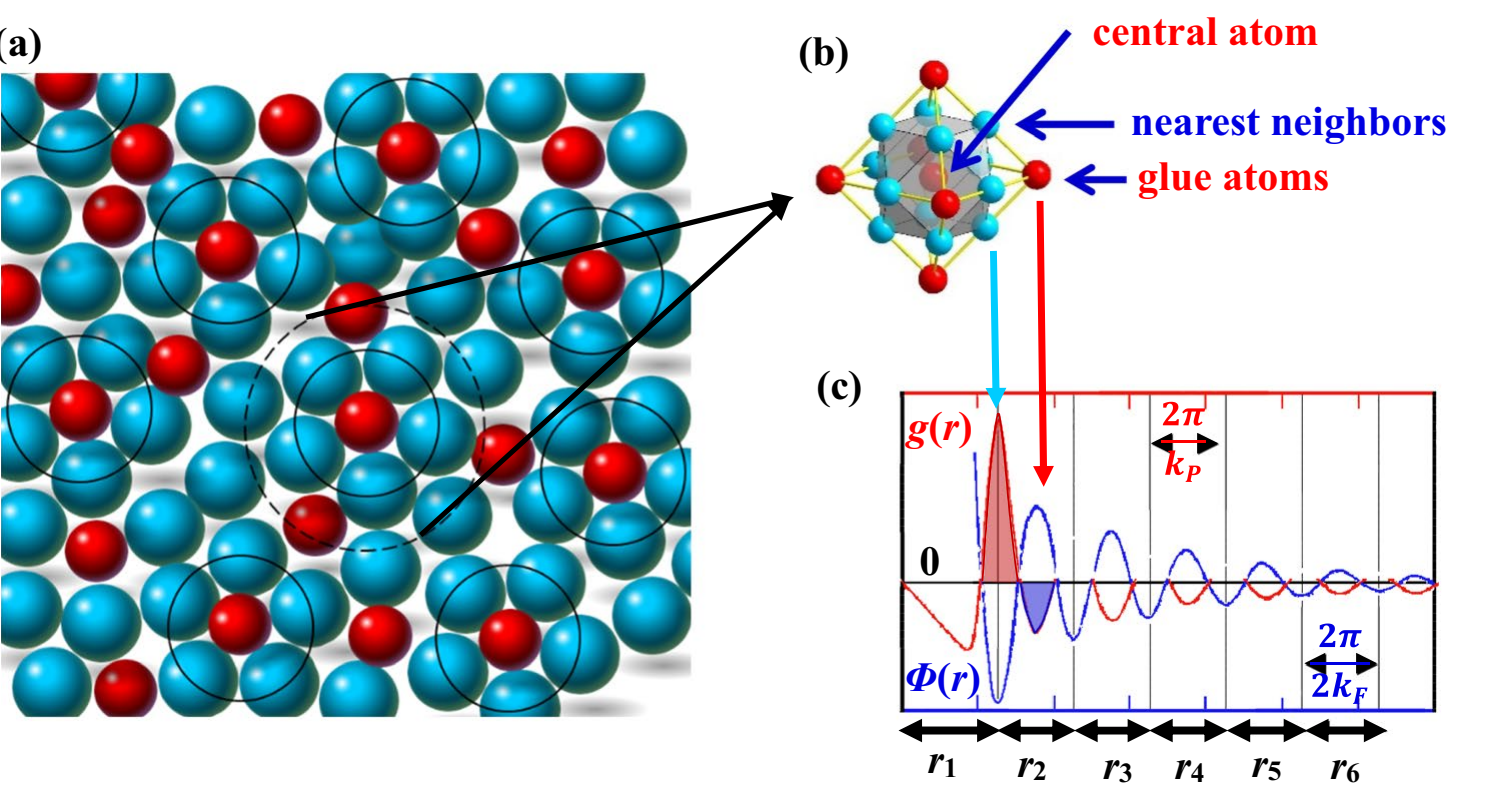
(**a**) Schematic diagram of short-range order and long-range disorder distribution of solute atoms in binary solid solution alloys. (**b**) Cluster configuration of binary FCC structure. (**c**) Idealized pair distribution function *g*(*r*) and total potential energy *Φ*(*r*) curve felt by electrons^12^.

We first briefly review the fundamentals that lead to chemical units, as fully detailed in reference^7^. Short-range ordering is formed due to the charge shielding around any given atom that produces oscillating distribution of electron density, namely Friedel oscillations^10,11^. As shown in Fig. 1c, the total potential function $\Phi (r{)} \propto {\text{ - sin(2}}k_{F} r{)/}r^{{3}}$ felt by the electrons at radial distance *r* periodically decays with the third power of *r*, where *k*_F_ is Fermi wave vector. This oscillating behavior of electrons in turn causes the same oscillation of atomic density *g*(*r*) in the real space, which is prominent in short *r* range, especially at the nearest and next-nearest neighborhoods. A local chemical unit is defined using a charge-neutral cut-off distance of 1.76*λ*_*Fr*_, *λ*_*Fr*_ = π/*k*_F_ being Friedel wavelength, that encloses the nearest-neighbor cluster and a few next-neighbor glue atoms. For FCC structure, its cluster-plus-glue-atom model is shown in Fig. 1b, the cluster is cuboctahedron with coordination number of 12 and the glue-atom shell in the next neighborhood is octahedron of coordination 6. A solid solution is then regarded as the random packing of such units as schematically illustrated in Fig. 1a. The chemical unit of a binary A–B system is expressed in cluster formula form as [A-M_12_]A_*x*_B_*y*_, where M_12_ = B_*n*1_A_12-*n*1_ refers to the average of nearest-neighbor atoms and integer *x* + *y* represents the number of glue atoms with 0 < *x* + *y* < 6.

Following^13^, the chemical unit volume is the sum of the each atomic volume $\left[ {(1 + x) \cdot R_{A}^{3} + 12 \cdot R_{M}^{3} + {\text{y}} \cdot R_{B}^{3} } \right] \cdot \left( {4\pi /3} \right)/0.74$, where *R*’s are atomic radii and 0.74 is the packing efficiency of FCC structure. This volume is also equal to the spherical volume enclosed by the charge-neutral cut-off distance 1.76*λ*_*Fr*_, $(4\pi /{3}) \cdot (1.76\lambda_{Fr} )^{3}$. Since *R*_A_ + *R*_M_ = 1.25 *λ*_*Fr*_ is the nearest-neighbor distance, the *x*–*y* relationship is obtained:

2 $$x \cdot R_{{\text{A/M}}}^{{3}} + y \cdot R_{{\text{B/M}}}^{{3}} \approx 2 \cdot \left( {R_{{\text{A/M}}} + 1} \right)^{3} - 12 - R_{{\text{A/M}}}^{3}$$

where *R*_A/M_ and *R*_B/M_ are respectively the ratios of *R*_A_ and *R*_B_ over $R_{M} = (n_{1} \cdot R_{B} + (12 - n_{1} ) \cdot R_{A} )/12$. Goldschmidt radii of atoms are generally adopted. When *R*_A_ = *R*_B_, *x* + *y* = 3, which means a 16-atom cluster formula for an FCC solid solution composed of solute and solvent atoms of equal atomic radii, or [A–B_12_](A,B)_3_.

As demonstrated in references^7,14,15^, compositions of commonly used industrial alloys such as Cu alloys, Al alloys, stainless steels, and Ni-based superalloys fall close to the model predictions, validating the presence of simple chemical units in metallic alloys and the generality of the cluster formulism. Our recent work^16^ shows that the model also applies in high-entropy alloys, after appropriate elemental classification. The solid solutions of hexagonal closed-packed type can be treated similarly, as it shows the same coordination number of 12 (the nearest-neighbor cluster is twinned octahedron) and is also close-packed. The body-centered cubic structure, featuring a rhombododecahedral cluster with a coordination number of 14 and a non-close-packing, should be dealt with separately, which is an on-going work.

Now we show the two basic procedures towards constructing the chemical unit with formula [A–B_*n*1_A_*c*1-*n*1_]A_*x*_B_*y*_ using the short-range-order parameter *α*_1_.

1. Determination of nearest-neighbor atoms using *α*_1_

For a given alloy with a known B’s atomic fraction *m*_B_ and coordination number *c*_*1*_, the number of B atoms in the nearest-neighbor shell, *n*_1_, is directly obtained by using the measured *α*_1_ value following Eq. (1):

3 $$n_{1} = m_{B} \cdot c_{1} \cdot \left( {1 - \alpha_{1} } \right)$$

The *n*_1_ value should be approximated into a nearby integer. When the short-range-order parameter *α*_1_ is negative, the integer is the roundup of *n*_1_, for B atoms tend to be enriched in the nearest neighbor shell due to the attractive interaction mode between the central A and neighboring B atoms. Alternative, when *α*_1_ is positive, the integer is the rundown of *n*_1_.

(2) Calculation of next-neighbor glue atoms via Eq. (2)

By introducing into Eq. (2) the atomic ratios *R*_A/M_ and *R*_B/M_, the relationship between *x* and *y* is established. This relationship should also agree with the alloy composition, i.e., (*n*_1_ + *y*)/(1 + *c*_1_ + *x* + *y*) = *m*_B_. For FCC, the (*x*, *y*) solution is also limited to 0 < *x* + *y* < 6. A unique set of (*x*, *y*) solution is then possible, from which two sets of close-integers are obtained, so that the measured alloy composition falls between the two chemical units.

These procedures will be detailed in analyzing typical examples of popular binary copper alloys in the next.

## Examples of binary Cu alloys

### Cu-30Zn alloy

Though industrial Cu–Zn binary alloys cover a Zn range up to ~ 40 wt%, Cu-30Zn, or cartridge brass, is the most widely used grade. The *α*_*i*_ parameters reaching a few tens of shells are accurately measured in a single crystal Cu_68.9_Zn_31.1_ by elastic neutron diffraction using ^65^Cu isotope over a wide reciprocal range^17^. All through the paper the subscript number after the element indicates atomic fraction or percentage and the number before the element is weight percentage. The measured *α*_1_ = − 0.137 indicates that the element in the cluster center site tends to be nearest-neighbored by the other element, which agree with the negative mixing enthalpy (Δ*H*_Cu-Zn_ = − 6 kJ/mol)^18^. In accordance with the general cluster formula of binary FCC solid solutions [A-M_12_]A_*x*_B_*y*_, solute Zn is placed in the cluster center and is nearest-neighbored by *c*_1_ = 12 atoms enriched in solvent Cu, leading to cluster formula [Zn-Cu_*n*1_Zn_12-*n*1_]Zn_*x*_Cu_*y*_ = [Zn-M_12_]Zn_*x*_Cu_*y*_, where M is the averaged nearest-neighbor atom.

First, the number of Cu atoms *n*_1_ in the nearest-neighbor shell is calculated by *α*_1_ following Eq. (3): *n*_1_ = 0.689·12·(1 + 0.137) = 9.40, which is further approximated into roundup integer 10 according to the negative interaction mode between Zn and Cu. Then the chemical unit becomes [Zn-Cu_10_Zn_2_]Zn_*x*_Cu_*y*_ with M = Cu_10/12_Zn_2/12_.

Second, the relationship between *x* and *y* is calculated by introducing *R*_Zn/M_ and *R*_Cu/M_ into Eq. (2): 1.23*x* + 0.96*y* = 4.53. The Goldschmidt atomic radii^19^ are *R*_Zn_ = 1.39 Å and *R*_Cu_ = 1.28 Å, *R*_M_ = (10*R*_Cu_ + 2*R*_Zn_)/12 = 1.30 Å, so that *R*_A/M_ = 1.39/1.30 = 1.07 and *R*_B/M_ = 1.28/1.30 = 0.98. In combination with the alloy composition, *m*_B_ = (10 + *y*)/(13 + *x* + *y*) = 0.689, the unique (*x*, *y*) solution is (2.3, 1.8). The close-integers are (2, 2) and (3, 1). The corrosponding chemical units are then [Zn-Cu_10_Zn_2_]Zn_2_Cu_2_ = Cu_12_Zn_5_ = Cu_70.59_Zn_29.41_ = Cu-30.01Zn (wt.%) and [Zn-Cu_10_Zn_2_]Zn_3_Cu_1_ = Cu_11_Zn_6_ = Cu_64.71_Zn_35.29_ = Cu-35.96Zn. The alloy composition Cu_68.9_Zn_31.1_ just falls between the two chemical unit compositions. The corresponding mass percentage 64.04–69.99 wt% Cu agrees eactly with the most widely used cartridge brass C26000 (nominal 70Cu–30Zn, with composition ranges specified being 68.5–71.5 Cu, 0.05 Fe max, 0.07 Pb max, 0.15 max other (total), bal Zn)^20^.

### Cu-8Al aluminum bronze

In Cu_85_Al_15_, *α*_1_ = − 0.17 is measured by X-ray diffuse scattering over an angular range from 8° to 60°^21^, which leads to *n*_1_ = 11.93 ≈ 12 and to chemical unit [Al-Cu_12_]Al_*x*_Cu_*y*_. Using *R*_Al_ = 1.43 Å and *R*_Al/Cu_ = 1.12, and complying to the alloy composition, the (*x*, *y*) solution is (1.69, 3.23) and the close integers are (2, 3) and (1, 4). The corrosponding chemical unit are [Al-Cu_12_]Al_2_Cu_3_ = Cu_15_Al_3_ = Cu_83.33_Al_16.67_ = Cu-7.83Al and [Al-Cu_12_]Al_1_Cu_4_ = Cu_16_Al_2_ = Cu_88.89_Al_11.11_ = Cu-5.04Al. The mass percentage of Al atoms range from 5.04 to 7.83, which explains the most popular C61000 (92Cu-8Al, with composition ranges being 6.0–8.5 Al, 0.05 Fe max, 0.02 Pb max, 0.20 Zn max, 0.10 Si max, 0.50 max other (total), bal Cu)^20^.

### Cu-20Ni alloy

In Cu_80_Ni_20_, the value of *α*_1_, as measured by neutron diffuse scattering using ^65^Cu isotope, is + 0.058^22^, which indicates the tendency of same element neighboring. The number of Cu atoms in the nearest-neighbor shell as calculated using *α*_1_ is 10 and the corresponding chemical unit is [Cu-Cu_10_Ni_*2*_]Cu_*x*_Ni_*y*_. Using *R*_Ni_ = 1.25 Å, the close-integer (*x*, *y*) solutions are (2, 1) and (1, 2), which corresponds to the chemical units [Cu-Cu_10_Ni_2_]Cu_2_Ni_1_ = Cu_13_Ni_3_ = Cu_81.25_Ni_18.75_ = Cu-17.57Ni and [Cu-Cu_10_Ni_2_]Cu_1_Ni_2_ = Cu_12_Ni_4_ = Cu_75.00_Ni_25.00_ = Cu-23.54Ni. The range from 17.57 to 23.54 of Ni atoms explains the alloy C71000 (80Cu-20Ni, specified ranges being 19–23 Ni, 0.05 Pb max, 1.00 Fe, 1.0 Zn max, 1.00 Mn, 0.5 max other (total), bal Cu), which is commonly used as condensers, condenser plates and electrical springs^20^.

### Cu-2Be beryllium bronze

In Cu_89.1_Be_10.9_ alloy, *α*_1_ =  + 0.077, as measured by X-ray diffusion scattering, which is indicative of same-element neighboring^23^. Within the framework of [Cu-M_12_]Cu_*x*_Be_*y*_, the number of Cu atoms *n*_1_ is 10.79 as calculated from *α*_1_ and is approximated into integer 11. Using M = Cu_11/12_Be_1/12_ and *R*_Be_ = 1.13 Å, the close-integer (*x*, *y*) solution are (2, 1) and (3, 0), leading to chemical units [Cu-Cu_11_Be_1_]Cu_2_Be_1_ = Cu_14_Be_2_ = Cu_87.50_Be_12.50_ = Cu-1.99Be and [Cu-Cu_11_Be_1_]Cu_3_ = Cu_15_Be_1_ = Cu_93.75_Be_6.25_ = Cu-0.94Be. This range explains alloy C17200 (1.8 to 2.0 Be, 0.20 Ni + Co min, 0.6 Ni + Co + Fe max, 0.10 Pb max, 0.5 max other (total), bal Cu), which is the most popular Cu-Be alloy for showing high strength and elasticity^20^.

It should be stressed that all the above alloy examples refer to the industrial grades the most popularly used in each alloy system. More examples are shown in Table 1, where most of the chemical units explain common industrial specifications. Exceptions are the formulas from alloys Ni_80_Cu_20_ and Ni_60_Cu_40_, which indicates that not all formulas correspond to good alloys but the reverse is true: popularly used industrial alloys always satisfy specific cluster formulas as this is required to reach solute homogenization states.

**Table 1:** Chemical units of typical binary solid-solution-based alloys with FCC structure, derived by combining the measured Cowley’s parameter *α*_1_ from Refs.^17,21–26^ and the cluster-plus-glue-atom model.

Exp. alloys at%	*α*_1_	Chemical units	wt% ranges	Alloys specifications
Cu_68.9_Zn_31.1_	− 0.137	[Zn-Cu_10_Zn_2_]Zn_2_Cu_2_–[Zn-Cu_10_Zn_2_]Zn_3_Cu_1_	64.0–70.0 Cu	C26000 (68.5–71.5 Cu) Cartridge brass
Cu_85_Al_15_	− 0.17	[Al-Cu_12_]Al_2_Cu_3_–[Al-Cu_12_]Al_1_Cu_4_	5.0–7.8 Al	C61000 (6.0–8.5Al) Aluminum bronze
Cu_80_Ni_20_	+ 0.058	[Cu-Ni_2_Cu_10_]Cu_1_Ni_2_–[Cu-Ni_2_Cu_10_]Cu_2_Ni_1_	17.6–23.5 Ni	C71000 (19–23 Ni)
Cu_89.1_Be_10.9_	+ 0.077	[Cu-Be_1_Cu_11_]Cu_2_Be_1_–[Cu-Be_1_Cu_11_]Cu_3_	0.9–2.0 Be	C17200 (1.80–2.0 Be) Beryllium bronze
Ni_77.5_Fe_22.5_	− 0.108	[Fe-Ni_11_Fe_1_]Fe_2_Ni_1_–[Fe-Ni_11_Fe_1_]Fe_1_Ni_2_	75.9–82 Ni	K14076 (75–78 Ni)
Ni_53.5_Fe_46.5_	− 0.077	[Fe-Ni_7_Fe_5_]Fe_2_Ni_1_–[Fe-Ni_7_Fe_5_]Fe_1_Ni_2_	51.2–57.5 Ni	N14052 (50.5 Ni min)
Fe_65_Ni_35_	− 0.051	[Ni-Fe_9_Ni_3_]Ni_2_Fe_1_–[Ni-Fe_9_Ni_3_]Ni_1_Fe_2_	32.3–38.7 Ni	K93601 (35–38)Ni Invar alloy
Fe_60_Ni_40_	− 0.058	[Ni-Fe_8_Ni_4_]Ni_2_Fe_1_–[Ni-Fe_8_Ni_4_]Ni_1_Fe_2_	38.7–45.0 Ni	K94490 (43.5–46.5 Ni)
Fe_50_Ni_50_	− 0.073	[Ni-Fe_7_Ni_5_]Ni_2_Fe_1_–[Ni-Fe_7_Ni_5_]Ni_1_Fe_2_	45.0–51.2 Ni	K94800 (47–49 Ni)
Ni_89_Cr_11_	− 0.055	[Cr-Ni_12_]Cr_1_Ni_2_–[Cr-Ni_12_]Ni_3_	5.6–11.2 Cr	NCr10 (9.0-10Cr)
Ni_80_Cu_20_	+ 0.08	[Ni-Cu_2_Ni_10_]Ni_2_Cu_1_–[Ni-Cu_2_Ni_10_]Ni_1_Cu_2_	20.0–26.5 Cu	
Ni_70_Cu_30_	+ 0.118	[Ni-Cu_3_Ni_9_]Ni_1_Cu_2_–[Ni-Cu_3_Ni_9_]Ni_2_Cu_1_	26.5–33.0 Cu	N04400, (28.0–34.0 Cu) Monel alloy 400
Ni_60_Cu_40_	+ 0.103	[Ni-Cu_4_Ni_8_]Ni_1_Cu_2_–[Ni-Cu_4_Ni_8_]Cu_3_	39.4–45.7 Cu	NCu40-2–1, (38.0–42.0 Cu)

The calculated weight percent compositions are comparable to certain alloy specifications. The grades in the table are all ASTM standard UNS numbers, except NCr10 and NCu40-2-1 which are GB/T 5235 standard of China.

It should be reminded that short-range order parameters such as Cowley’s α parameter are sensitive to processing parameters, especially temperature^27^. In principle, the short-range-order parameters should be measured in alloys annealed near the critical temperature where long-range order disappears completely and the atomic distribution tends to be stochastically stable^28,29^. However, the critical temperature is usually unknown in a given alloy. Therefore, the measured α parameters should be more appropriately taken as the tendency along which atoms partition between the nearest-neighbor sites and the next-neighbor glue sites within the molecule-like chemical unit. This is why, for example, Cu-30Zn brass can also be linked to the cluster formula [Zn-Cu_12_]Zn_4_ as we previously proposed^7^, which can be regarded as the extreme case when the negative interaction model between Zn and Cu is fully complied, though this formula is equivalent to [Zn-Cu_10_Zn_2_]Zn_2_Cu_2_ as calculated from the measured α_1_.

Finally it should be emphasized that the present work is a combination of our theoretical model with measurable parameters such as the well-established Cowley’s α_1_. This endeavor strengthens the capability of our model in interpreting alloy compositions. However, the approach developed in the present work cannot be readily extended to multi-component systems (here we confine ourselves to binary systems only), where both the theoretical description and the experimental measurement on short-range ordering are highly difficult. It is noted that, during the last decade, research on short-range ordering is reviving, especially in high-entropy alloys^30–33^. The information provided by sophisticated measuring techniques and by computer simulation will surely enrich our knowledge on chemical short-range ordering. It should be our future goal to use the up-to-date data to deal with composition-complex alloys.

## Conclusions

To summarize, after combining the measured short-range-order parameters with our cluster-plus-glue-atom model, we are able to construct molecule-like chemical units which interpret existing industrial alloy composition as specified by standard grades. This work answers the long-standing question on the composition origin of solid-solution-based industrial alloys, by tracing to the molecule-like chemical units implied in chemical short-range ordering in solid solutions.

## Data Availability

The authors declare that the main data supporting the findings of this study are contained within the paper. All other relevant data are available from the corresponding author upon reasonable request.

## References

[1] Bruni G (1925). Solid solutions. Chem. Rev..

[2] Bragg WH (1922). The significance of crystal structure. J. Chem. Soc. Trans..

[3] Bragg WL, Williams EJ (1934). The effect of thermal agitation on atomic arrangement in alloys. Proc. R. Soc. Lond. A.

[4] Bethe HA (1935). Statistical theory of superlattices. Proc. R. Soc. Lond. A.

[5] Cowley JM (1950). An approximate theory of order in alloys. Phys. Rev..

[6] Dong C (2007). From clusters to phase diagrams: Composition rules of quasicrystals and bulk metallic glasses. J. Phys. D..

[7] Dong D, Wang Q, Dong C, Nieh T (2021). Molecule-like chemical units in metallic alloys. Sci. China Mater..

[8] Jiang B, Wang Q, Dong C, Liaw P (2019). Exploration of phase structure evolution induced by alloying elements in Ti alloys via a chemical-short-range-order cluster model. Sci. Rep..

[9] Harrison W (1970). Solid State Theory.

[10] Friedel J (1954). Electronic structure of primary solid solutions in metals.. Adv. Phys..

[11] Friedel J (1958). Metallic alloys. Nuovo Cimento.

[12] Häussler P (1992). Interrelations between atomic and electronic structures-liquid and amorphous metals as model systems. Phys. Rep..

[13] Han G (2011). The e/a values of ideal metallic glasses in relation to cluster formulae. Acta Mater..

[14] Chen C, Wang Q, Dong C, Zhang Y, Dong H (2020). Composition rules of Ni-base single crystal superalloys and its influence on creep properties via a cluster formula approach. Sci. Rep..

[15] Wen D (2018). Developing fuel cladding Fe-25Cr-22Ni stainless steels with high microstructural stabilities via Mo/Nb/Ti/Ta/W alloying. Mater. Sci. Eng. A.

[16] Ma Y (2021). A novel soft-magnetic B2-based multiprincipal-element alloy with a uniform distribution of coherent body-centered-cubic nanoprecipitates. Adv. Mater..

[17] Reinhard L, Schönfeld B, Kostorz G, Bührer W (1990). Short-range order in α-brass. Phys. Rev. B.

[18] Takeuchi A, Inoue A (2005). Classification of bulk metallic glasses by atomic size difference, heat of mixing and period of constituent elements and its application to characterization of the main alloying element. Mater. Trans..

[19] Gaskell P (1981). Acta Metall.

[20] Davis, J. R. *ASM Handbook Volume 2: Properties and Selection: Nonferrous Alloys and Special-Purpose Materials* (ASM International, 1998).

[21] Kulish N, Petrenko P (1990). Short-range order in binary solid solutions. Ordering and its change on heating in Fe-Al, Cu-Al, and Ag-Al alloys. Phys. Status Solidi A.

[22] Vrijen J, Radelaar S (1978). Clustering in Cu-Ni alloys: A diffuse neutron-scattering study. Phys. Rev. B..

[23] Koo Y, Cohen J, Shapiro S, Tanner L (1988). AS-quenched Cu-10.9 at% Be. Acta Metall..

[24] Schweika W, Haubold H (1988). Neutron-scattering and Monte Carlo study of short-range order and atomic interaction in Ni_0.89_Cr_0.11_. Phys. Rev. B..

[25] Bokoch SM, Tatarenko VA (2010). Interatomic interactions in FCC Ni-Fe alloys. Usp. Fiz. Met..

[26] Jiang X, Ice GE, Sparks CJ, Robertson L, Zschack P (1996). Local atomic order and individual pair displacements of Fe_46.5_Ni_53.5_ and Fe_22.5_Ni_77.5_ from diffuse x-ray scattering studies. Phys. Rev. B..

[27] Zhang R (2020). Short-range order and its impact on the CrCoNi medium-entropy alloy. Nature.

[28] Cowley JM (1960). Short-and long-range order parameters in disordered solid solutions. Phys. Rev..

[29] Sadigh B (1999). Short-range order and phase stability of surface alloys: PdAu on Ru (0001). Phys. Rev. Lett..

[30] Singh P, Smirnov AV, Johnson DD (2015). Atomic short-range order and incipient long-range order in high-entropy alloys. Phys. Rev. B..

[31] Chen X (2021). Direct observation of chemical short-range order in a medium-entropy alloy. Nature.

[32] Singh R, Sharma A, Singh P, Balasubramanian G, Johnson DD (2021). Accelerating computational modeling and design of high-entropy alloys. Nat. Comput. Sci..

[33] Kostiuchenko, T., Ruban, A. V., Neugebauer, J., Shapeev, A. & Krmann, F. Short-range order in face-centered cubic VCoNi alloys. *Phys. Rev. Mater.* **4**, 113802 (2020).

